# Nutritional Composition, In Vitro Antioxidant Activity and Phenolic Profile of Shortcrust Cookies Supplemented by Edible Flowers

**DOI:** 10.3390/foods10112531

**Published:** 2021-10-21

**Authors:** Kristýna Šťastná, Daniela Sumczynski, Erkan Yalcin

**Affiliations:** 1 Faculty of Technology, Tomas Bata University in Zlín, Náměstí T.G. Masaryka 5555, 76001 Zlín, Czech Republic; kstastna@utb.cz; 2 Faculty of Engineering, Gölköy Campus, Bolu Abant Izzet Baysal University, 14030 Bolu, Turkey; yalcin_e@ibu.edu.tr

**Keywords:** antioxidant capacity, biscuit, photochemiluminiscence assay, phenolic acid, flavonoid, flower, free phenolic, bound phenolic

## Abstract

In this study, the changes in nutritional composition, phenolic compounds and antioxidant activity in free and bound fractions of shortcrust cookies were investigated. By incorporating ingredients such as kamut, matcha tea, dried mango and jasmine flowers, the contents of crude and neutral-detergent fibre reached up to 2.0% and 5.0%, respectively. Similar increments were observed in phenolic compound contents and 2,2-diphenyl-1-picrylhydrazyl scavenging activity values. Concerning cookies supplemented with matcha tea, the total phenolic compound content raised from 1.0 to 4.8 mg gallic acid equivalent/g and the antioxidant activity value increased from 0.5 to 5.7 mg trolox equivalent/g on a dry weight basis. For determining the antioxidant activity values in water-soluble and insoluble phenolic fractions of the cookies, a photochemiluminiscence assay was separately applied, and they were found up to 0.8 mg ascorbic acid equivalent/g and 3.2 mg trolox equivalent/g, respectively. The main phenolic compounds in all supplemented cookies were neochlorogenic, gallic and vanillic acids.

## 1. Introduction

Short dough butter cookies are popular snack products with long shelf lives and a delicate taste, but they should not be the main source of energy and antioxidants in our diet because they usually contain relatively high sugar and fat content and ingredients with low contents of biologically active substances. This mostly concerns cookies with chocolate covering or chips; cookies without these ingredients are considered to be healthier [1,2].

The main ingredients are usually wheat flour, butter and sucrose. In the market, we can usually find cookies flavoured with vanilla, caramel, ginger, cinnamon, cocoa, fruits or nuts [1], but consumers in developed countries are looking for novel tastes and highly nutritious cookies. Wheat flour can be easily replaced with different flours rich in protein or dietary fibre, because rheological properties of wheat gluten in this type of product is not crucial since high contents of fat and sugars prevent its development during mixing [1,3]. Flour can also be supplemented with pulses or oilseeds flours in order to enrich them with protein, fat soluble vitamins, antioxidants or minerals [4].

An enriching of cookies with edible flowers can be an alternative approach in search of new flavours. Some well-liked flowers include rose, violet, pansy or daylily, but not all of them are suitable for cookie production because of their bitter taste, e.g., chrysanthemum [5]. The higher concentrations of different bioactive compounds with antioxidant activities exhibiting many valuable health effects on humans is the main reason for the application of edible flowers in cookie production [5,6]. It is generally known that phenolics are compounds that mainly provide antioxidant, anti-inflammatory or anti-proliferative properties [5]. In the case of bioactive compounds, the composition of phenolics depends on the species of edible flower [7]. They are usually rich sources of flavonoids [5], mainly kaempferol and quercetin. Edible flowers, e.g., *Centaurea cyanus*, are also a good source of phenolic acids, such as caffeic and *p*-coumaric derivatives [7,8]. Anthocyanin and carotenoid derivatives are responsible for their colour, and flowers of *Calendula officinalis* contain up to 7200 μg/g total carotenoids [6,7]. Radical scavenging activity measurement for the determination of antioxidant activity is strongly correlated to the presence of wide ranges of phytochemicals [9]. Regarding these potential nutritional benefits of non-traditional ingredients, Tomas Bata University in Zlín published utility model No. 33 013 (2019) at the Industrial Property Office of the Czech Republic where edible flowers were applied to the production of biscuits and durable pastries.

There are a number of studies that focus on the content of bioactive substances in individual ingredients; however, there is lack of studies that focus on the overall evaluation of their use. Therefore, the aim of this study was to evaluate the impact and significance of the addition of a combination of non-traditional cereal flours, dry fruit and edible flowers on chemical composition (ash, crude protein, starch and crude fat contents) and nutritional properties (phenolic compound and antioxidant activity) of short dough butter cookies. The basic chemical composition of the cookies was complemented with the measurement of crude fibre (CF) and neutral-detergent fibre (NDF) contents and in vitro digestibility values. Total phenolic compounds (TPC), free and bound phenolic compounds were also measured using high pressure liquid chromatography (HPLC). Radical scavenging antioxidant activity was measured spectrophotometrically using ABTS and DPPH radicals. In addition, the photochemiluminiscence (PCL) assay was performed to measure the lipid- (ACL) and water (ACW)-soluble antioxidant compounds of the cookies.

## 2. Materials and Methods

### 2.1. Materials

Substitute flours, such as whole spelt flour, oat flakes, barley flakes, kamut flour and red wheat, were purchased from local markets (Zlín, Czech Republic). Oat and barley flakes were milled using a Combistar mill (Waldner Biotech, Lienz, Austria) to a particle size of about 200 µm. Dried edible flowers, such as lavender buds *(Lavandula angustifolia)*, rose petals *(Rosa centifolia)*, hop bracts *(Humulus lupulus)*, jasmine buds (*Jasminum officinale)* and elderflower inflorescence *(Sambucus nigra)*, were provided by local flower producers (Sonnentor, Hodonín, Czech Republic; Lbros, Vrchlabí, Czech Republic). Flavouring ingredients, such as sulphured dried apricot, dried malt extract, unsweetened dried mango slices, dried lemon peel powder, unsweetened dried ginger, unsweetened dried apple, lyophilised raspberry and strawberry fruits, cashew nut and matcha tea as well as butter (80 % of milk fat), beetroot powder sugar, fresh egg yolk, vanillin sugar and salt, were also purchased from local markets (Zlín, Czech Republic).

### 2.2. Chemicals and Reagents

Neutral-detergent fibre (NDF) was determined using a solution package containing sodium lauryl sulphate, EDTA disodium, sodium borate and sodium phosphate dibasic together with glycol and *α*-amylase (all Ankom Technology, NewYork, NY, USA). To study digestibility, pepsin (0.7 FIG-U/mg) and a mixture of pancreatin enzymes (350 FIG-U/g protease, 6000 FIG-U/g lipase and 7500 FIG-U/g amylase) were used (all Merck, Darmstadt, Germany). To determine antioxidant activities, 2,2′-azinobis(3-ethylbenzo-thiazoline-6-sulfonic acid) diammonium salt (ABTS), 2,2-diphenyl-1-picrylhydrazyl (DPPH) and standard 6-hydroxy-2,5,7,8-tetramethylchroman-2-carboxylic acid (trolox) (Sigma-Aldrich, St. Louis, MO, USA) were used. PCL was measured and evaluated using ACL and ACW kits supplied by Analytik Jena AG (Jena, Germany). For the determination of phenolic profile, the standard compounds (epigallocatechin, catechin, epicatechin, rutin, quercetin, kaempferol, neochlorogenic, chlorogenic, gallic, protocatechuic, *p*-hydroxybenzoic, vanillic, caffeic, syringic, *p*-coumaric, ferulic, sinapic, ellagic, *o*-coumaric, cinnamic acids and protocatechuic acid ethyl ester) were purchased from Sigma-Aldrich, St. Louis, MO, USA. All standards and solvents were HPLC-grade (purity ≥ 98.5%).

### 2.3. Preparation of Shortcrust Cookies

**Figure 1:**
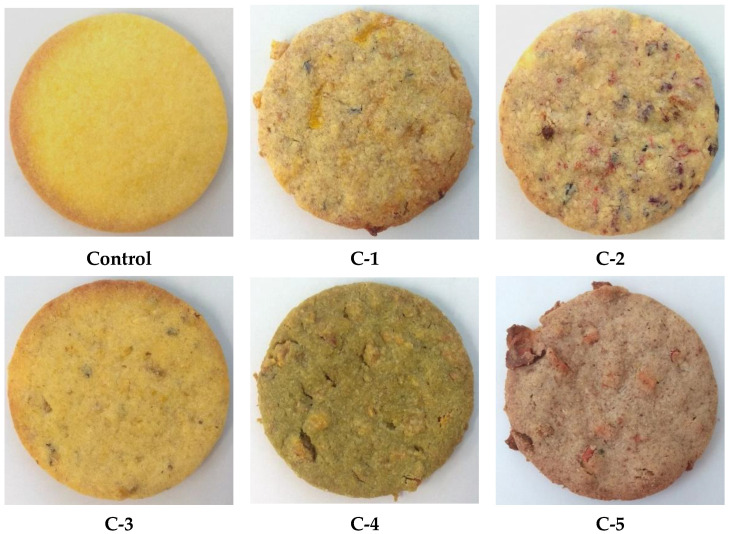
Baked cookies supplemented by non-traditional flours and edible flowers according to recipe presented in Table 1.

Six types of shortcrust cookies (Figure 1) were prepared in this study, including a cookie prepared with wheat flour used as the control. The other five cookie samples were prepared with different cereal flours and flavouring ingredients such as dried fruit, nuts and edible flowers (samples C-1 to C-5).

Detailed cookie recipes are presented in Table 1. Differences in the percentage of basic ingredients (flour, butter, powdered sugar and egg yolk) are caused by the need to process the dough for the subsequent shaping of the cookie. These were prepared in amounts of 1000 g and chilled for 24 h at 8 °C. After this step, the dough was sheeted to a thickness of 3 mm and sliced using a cookie cutter with a diameter of 5 cm. This unbaked product was chilled again for 10 min at 8 °C and then was baked in an oven at 175 °C for about 6.5 min. Baked cookies were cooled to laboratory temperature and stored in vacuum-sealed bags in an air-conditioned and sunless room for further analyses, which were carried out within two weeks.

### 2.4. Chemical Composition Analysis of the Cookies

The determination of dry matter (DM) and ash contents were carried out gravimetrically [10,11]. Total nitrogen content was measured according to the Kjeldahl method; crude protein (CP) content was then calculated (N%*6.25). Crude fat (CFat) content was determined following the Soxhlet method according to AACC Method No. 30-25.01 [12]. Starch content was determined using the Ewers’ polarimetric method [13]. Total carbohydrates (TCH) were calculated following the equation below [14]:

(1) TCH(%)=100−(CFat%+CP%+Ash%)

Total energy value of the cookie was calculated by the equation described below [14]:

(2) Energy (kcal/100 g)=(4×g CP)+(4×g TCH)+(9×CFat)

The contents of CF and NDF were determined following the methods of Sumczynski et al. and the results were calculated as follows [15]:

(3) $$\mathrm{CF}\;\mathrm{or}\;\mathrm{NDF}=\frac{\left(m_{3}-m_{1}c_{1}\right)-\left(m_{4}-m_{1}c_{2}\right)}{m_{2}}\times 100$$

(4) $$c_{1}=\frac{m_{B}}{m_{1}}$$

(5) $$c_{2}=\frac{m_{A}}{m_{1}}$$

where CF or NDF: content of fibre (%); m_1_: weight of extraction bag (g); m_2_: weight of sample (g); m_3_: weight of bag with residue of sample after hydrolysis (g); m_4_: weight of ash after burning the bag with residue of sample after hydrolysis (g); c_1_: correction of weight of bag after hydrolysis (-); c_2_: correction of weight of sample after combustion (-); m_B_: weight of bag after hydrolysis (g); m_A_: weight of bag ash after combustion (g).

### 2.5. In Vitro Digestibility Assay

The in vitro digestibility of the cookies was measured using the method of Mišurcová et al. [16]. A homogenised sample was weighted into extraction F57 bags (Ankom Technology, New York, NY, USA) in amounts of 0.25 g. Bags were sealed and one empty bag was used for correction. Bags were incubated (Daisy Incubator, Ankom Technology, New York, NY, USA) in 1700 mL of 0.1 M HCl, including 3 g pepsin at 40 °C for 4 h. Then, bags were washed in distilled water and incubation was repeated in 1700 mL of phosphate buffer (pH 7.45), including 3 g pancreatin at 40 °C for 24 h. To precipitate starch, the phosphate buffer tank with bags was heated at 80 °C for 40 min. After this step, bags were washed again in distilled water, dried at 103 °C for 24 h and burned at 550 °C for 5.5 h. Dry matter digestibility (DMD, %) and organic matter digestibility (OMD, %) values were calculated using the following equations:

(6) $$\mathrm{DMD}=100-\frac{100\times \mathrm{DRM}}{m_{2}\times \mathrm{DM}}$$

(7) $$\mathrm{DMR}=m_{3}-m_{1}c_{1}$$

(8) $$\mathrm{DM}=\frac{\mathrm{dm}\times m_{\mathrm{dm}}}{100}$$

(9) $$\mathrm{OMD}=100-\frac{100\times \left(\mathrm{DMR}-\mathrm{AR}\right)}{m_{2}\times \mathrm{DM}\times \mathrm{OM}}$$

(10) $$\mathrm{AR}={\;m}_{4}-m_{1}c_{2}$$

(11) $$\mathrm{OM}=\frac{\mathrm{dm}-\mathrm{ash}}{100}$$

(12) $$c_{1}=\frac{m_{5}}{m_{1}}$$

(13) $$c_{2}=\frac{m_{6}}{m_{1}}$$

where DMR: weight of sample after incubation (g); DM: dry matter of sample (g); dm: dry matter of sample (%); AR: ash content of sample (g); ash: ash content of sample (%); OM: organic matter of sample (g); m_1_: weight of empty bag (g); m_2_: weight of sample (g); m_3_: weight of dried bag with sample after incubation (g); m_4_: weights of both sample and sack after drying and combustion (g); m_dm_: weight of sample for dry matter determination (g); c_1_: weight correction of empty bag after incubation (g); c_2_: weight correction of empty bag after combustion (g); m_5_: weight of empty bag after incubation (g); m_6_: weight of ash after burning empty bag (g).

### 2.6. Extraction of Phenolic Compounds of the Cookies

#### 2.6.1. Defatting of Cookie Samples

The samples were defatted by using the method of Alrahmany et al. with some modifications [17]. A homogenised sample (8 g) was mixed with hexane in a ratio of 1:5 (*w*/*v*) on a magnetic stirrer for 1 h. These mixtures were centrifuged (Dynamica Scientific Ltd., Newport Pargnell, UK) at 12,300× *g* for 15 min. Hexane was decanted and the precipitate was quantitatively poured into a Petri dish using absolute ethanol and dried at 40 °C until a constant weight was achieved. The difference between the sample weight and the defatted sample was used to convert total phenolic content, antioxidant activity and the profile of individual phenolic contents to the original weight of the sample.

#### 2.6.2. Extraction of Free Phenolic Compounds

Free and bound phenolics were extracted using the method by Kotásková et al. with minor modifications [18]. The defatted sample was weighed twice to 2 g into dark flasks and 8 mL of 80% methanol and 1 drop of ethyl acetate were added into each flask. The mixtures were put into the ultrasonic bath for 1 h and then they were centrifuged at 12,300× *g* for 15 min. Supernatants were used as an extract of free phenolics and the precipitate was kept for the extraction of insoluble bound phenolics.

#### 2.6.3. Extraction of Bound Phenolics

The precipitate was washed with 20 mL of distilled water. Then, 25 mL of 0.1 M NaOH was added and left in ultrasonic bath for 1 h. The mixture was centrifuged at 12,300× *g* for 15 min. Then, the pH of supernatant was adjusted to the range of pH 3–5 using 6 M HCl and centrifuged again at 12,300× *g* for 15 min. This supernatant was used as an extract of insoluble bound phenolics.

### 2.7. Determination of Total Phenolic Compound Content

Extracts of free and insoluble bound phenolic compounds were directly used in total phenolic compound (TPC) contents determination using the spectrophotometric method of Folin–Ciocalteu reagent [19]. For this purpose, 5 mL of distilled water, 200 μL of extract, 0.5 mL of Folin–Ciocalteu reagent and 1.2 mL of 20% Na_2_CO_3_ were mixed and then the volume was completed to 10 mL with distilled water. After incubation in the dark for 30 min, the absorbance was measured at 765 nm (Specord 50 PLUS, Analytic Jena AG, Jena, Germany). Gallic acid (0 to 800 mg/L) was used to create the reference standard line in order to calculate the sample concentrations. The results were expressed as mg of gallic acid equivalent per g of dry sample.

### 2.8. Antioxidant Activity Measured Using DPPH and ABTS Radicals

Antioxidant activity values of the cookies’ free and bound extracts were assessed by quenching of DPPH and ABTS radicals, in accordance with Sumczynski et al. [20]. To determine the DPPH radical scavenging activity, 4 mL of working DPPH solution and 210 μL of extract were mixed in a glass tube. After incubation in the darkness for 60 min, the absorbance at 515 nm was measured using the spectrophotometer. To determine the quenching of ABTS radical, 4 mL of working ABTS solution and 50 μL of extract were mixed in a glass tube. After incubation in darkness for 30 min, the absorbance at 734 nm was measured. The reference standard line was prepared according to different concentrations of trolox (0 to 180 mg/L) corresponding to absorbance values. The results were expressed as mg of trolox equivalent per g of dry sample.

### 2.9. Determination of Individual Phenolics Using HPLC

Individual phenolics in free and bound extracts of the cookie samples were determined using an HPLC-DAD (Thermo Scientific Dionex Ultimate 3000, DAD-300 RS, Waltham, MA, USA) according to Kotásková et al. with minor modifications [18]. To separate individual phenolics, a Kinetex C18 column (150 × 4.5 mm; 2.6 µm, Phenomenex, Torrance, CA, USA) was used. For the analysis, 10 μL of extract was injected into the system with a flow rate of 1 mL/min, and the column temperature was set at 30 °C. The following conditions for mobile phases were used: redistilled water:acetic acid in a ratio of 99:1 (A) and redistilled water:ACN:acetic acid in a ratio of 67:32:1 (B) with the gradient program of 10% B at 0 min; 20% B between 0 and 10 min; 20–40% B between 10 and 16 min; 40–50% B between 16 and 25 min; 50–70% B between 25–26 min; 70% B between 26 and 30 min; 70–10% B between 30 and 40 min, and 10% B between 40 and 45 min. The gained chromatograms were recorded at 275 mm. The DAD response was linear for all phenolic standards within the range of 0 to 50 μg/mL with correlation coefficients exceeding 0.9995. Individual phenolics were identified according to their retention time obtained from the chromatogram of the corresponding standards.

### 2.10. Antioxidant Activity Determination Using Photochemiluminiscence Assay

The photochemiluminiscence (PCL) determination was carried out according to Besco et al. using ACW and ACL kits and Photochem device from Analytic Jena AG (Jena, Germany). This assay involves the photochemical generation of superoxide radicals combined with chemiluminescence detection. The PCL method allows one to evaluate the antioxidant capacity of both lipophilic (ACL) and hydrophilic (ACW) compounds [21].

In the first step, homogenised cookie samples (2.0 g) were separately extracted with 5 mL of redistilled water (ACW) or 5 mL of methanol (ACL). This mixture was placed in a shaking water bath at 40 °C for 30 min and centrifuged at 12,300× *g* for 15 min and filtered through a 0.22 μm nylon syringe filter. The obtained extracts were immediately diluted with reagent 1 of the ACW and ACL kits and measured by Photochem to evaluate the antioxidant potential of water- (ACW) and lipid-soluble (ACL) antioxidant compounds.

The results are expressed as mg of ascorbic acid equivalent (AAE/g) of dry sample for ACW and mg of trolox equivalent (TE/g) of dry sample for ACL. For the preparation of standard solutions, ACW and ACL protocols were applied. Reagent 1 in an amount of 490 µL (kit ACW) and 10 µL of H_2_SO_4_ were added to the vial containing ascorbic acid (reagent 4, kit ACW) and mixed for 30 s. This solution was diluted with reagent 1 to obtain the work solution, where 10 µL contained 1 nmol of ascorbic acid, used as a calibration standard. For the preparation of a trolox standard, 500 µL of reagent 1 (kit ACL) was added into vial with trolox (reagent 4, kit ACL) and mixed for 20 s to obtain stock solution that was then diluted with reagent to the concentration of 1 nmol of trolox in 10 µL of this solution. Measurements of each standard were repeated five times.

### 2.11. Statistical Analysis

All analyses were repeated 5 times and the results are reported as the mean ± standard deviation (SD) on a 100 g of dry weight basis. The results were statistically evaluated using one-way analysis of variance (ANOVA). Tukey’s test was applied to identify differences among means. The level of confidence was set to 95% (*p* < 0.05) and correlation between the data was defined using Pearson’s correlation coefficient (*r*).

## 3. Results

### 3.1. Chemical Analysis

The chemical compositions of non-traditional shortcrust butter cookies are shown in Table 2. The dry matter content of the cookies was in the range of 94.7–98.0%, which meets the conditions for extended shelf-life, since the moisture content should be below 10%. The CP content ranged from 8.4 to 11.0%. It can be seen that the presence of flavouring ingredients decreases the amount of CP contents in the samples of C-3, C-4 and C-5. The protein content of wheat flour is in the range of 12–15% [22]; on the other hand, the average protein contents of oat and barley are reported as 12% and 10–17%, respectively [23,24].

The CFat contents of the cookies changed from 22.6 to 30.8% due the fluctuating amount of butter and egg yolk used in the formulation fluctuated. Although shortening is usually used as a dough lubricant [3], butter was used as the texture and sensory developer. Despite the fact that the physico-chemical properties of butter are not stable during storage, it has some advantages, e.g., a high amount of easily metabolised short-chain fatty acids and good sensory properties appear during the short shelf life due to the presence of lipases [25]. Only the sample C-2 contained a higher amount of CF than the control sample due the presence of cashew nuts in the formulation, since they contain a proportion of up to 51% crude oil [26].

The main constituent of cookies was TCH, which changed from 61.1 to 72.5%, with starch as the leading carbohydrate, ranging from 29.3 to 43.6%. This range is the result of different amounts of starch in formulations and different starch contents in those substituting flours. The control sample had the highest starch content (43.6%); on the other hand, the sample C-4 had the highest carbohydrate content (72.5%). The starch content of wheat flour was reported in the range of 60–80% [27], the starch content of oats was up to 66% [28], and whole barley meal had starch content around 65% [24].

The ash, CF and NDF contents of the cookies increased compared to the control sample. Different substituting flours and flavouring ingredients were the main reasons for these increments. The highest ash content was found in the sample C-1 as 1.44% due the presence of the wholegrain spelt flour, which increased the ash content 2.5 times compared to the control. The highest CF and NDF contents were presented in C-4 as 2.00 and 4.99%, respectively. Those higher contents are the result of using high-fibre ingredients such as kamut flour, unsweetened dried mango and matcha tea in the formulation. It is known that CF includes cellulose and lignin, and NDF includes cellulose, lignin and insoluble hemicelluloses [15].

Energy values of the cookies ranged from 2110 (C-4) to 2282 kJ/100 g (C-2). The latter was the only sample with an energy value that increased compared to the control sample (2245 kJ/100 g). This increase is caused by the presence of cashew nuts that also increase the CFat content. The main contributor to energy values in all samples was butter, which constituted up to 28% of the sample composition. The sample C-4 had the lowest energy value (2110 kJ/100 g) because it had the highest CF and NDF and the lowest CFat contents among the cookies.

OMD and DMD values of the cookies ranged from 95.3 to 99.2% and from 94.1 to 98.3%, respectively. The amount of CF had an impact on OMD and DMD values, as observed in Table S1, where the CF content of the cookies exhibited the highest correlations (*r* = 0.9530 and 0.9462) with DMD and OMD, respectively. It means that a higher CF value results in lower OMD and DMD values. This is an important finding for health, since the blood glucose level will not increase when the consumers have high-fibre cookies. Similar relationships between NDF content and digestibility values were determined in this study.

### 3.2. Phenolic Compound Contents and Antioxidant Activity Values of the Cookies

Phenolic compound contents and antioxidant activity (AOA) values of the cookies in free and bound phenolic fractions are shown in Table 3. The antioxidant activity values measured using PCL method are also shown in Table 3.

Total phenolic compound (TPC) contents of the cookies significantly increased with the addition of flavouring ingredients, from the 1.01 mg GAE/g value in control sample to 4.76 mg GAE/g in the sample C-4 which also had the highest free phenolic compound content (3.24 mg GAE/g). Compared to the control sample, the free TPC value assessed in the sample containing matcha was almost seven times higher as a result of the combination of matcha powder that is usually high in phenolics, especially in catechin antioxidants. For instance, matcha contains around 200 mg GAE/g in free phenolic compounds [29]. Not only matcha tea, but also mango is a valuable source of phenolics [30]. Regarding bound fractions, the highest bound phenolic compound content (1.56 mg GAE/g) was observed in the sample C-2 that contained rose and berry fruits. The free phenolic compound contents of the cookies were higher than those of the bound ones, except for the control sample.

The scavenging activities of DPPH and ABTS radicals were used to measure the antioxidant activity of the cookies. In total, the DPPH scavenging activity ranged from 0.47 (control) to 5.67 mg TE/g (C-4). Similar results were obtained using ABTS radical, where the lowest and the highest antioxidant activity values ranged from 1.25 (control) to 13.6 mg TE/g (C-4). The distribution of antioxidant activity values in free and bound phenolic compounds varied among cookies. The highest antioxidant activity value among bound phenolics was found in sample C-2 as 1.12 mg TE/g and 3.74 mg TE/g for scavenging of DPPH and ABTS, respectively. The antioxidant activity of control sample arose from cereal flour, where phenolics bound to cell wall components were mainly responsible [31].

Generally, the scavenging activities had positive correlations with free, bound and total phenolic compound contents (Table 4). For instance, the correlations were 0.9993 and 0.9899 between DPPH scavenging activity and TPC content and ABTS scavenging activity and TPC contents of the cookies, respectively. The correlations between scavenging activities and free/bound phenolic were strong positive with some fluctuations.

The results of ACW and ACL are also shown in Table 3. It is stated that ACW represents the antioxidant capacity of water-soluble compounds, e.g., ascorbic acid and flavonoids, and ACL stands for the antioxidant capacity of lipid-soluble compounds, e.g., tocopherols, tocotrienols and carotenoids [21]. In case of ACW, the results ranged from 0.04 (control) to 0.81 mg AAE/g (C-5). The sample of C-5 had a 20.3× higher ACW value than the control sample, since its recipe included red wheat as a substitute for flour and elderflower and also dried apple and dried lemon peel flavouring ingredients, which were good sources of flavonoids and vitamin C [32]. The ACL results of all cookies were higher than those of the ACW results. The ACL results of cookies ranged from 0.06 (control) to 3.19 mg TE/g (C-4). The recipe of the C-4 cookie included Kamut flour as a substituting flour and jasmine flower, mango and matcha tea as flavouring ingredients. It was thought that the chlorophylls of matcha tea may be the main contributor to the ACL value. Similarly, the high ACL value of the C-2 cookie (1.39 mg TE/g) can be explained by the presence of oat flakes with germ, which contain up to 1.8 mg of tocopherols and tocotrienols per 100 g of grains [33].

### 3.3. Flavonoid, Stilbene and Phenolic Acid Contents of Free and Bound Phenolic Compounds

The contents of seven flavonoids and one stilbene compound investigated in cookies are shown in Table 5. The highest total catechin (46.7 µg/g), kaempferol (7.84 µg/g) and quercetin (3.78 µg/g) contents were determined in the cookies of C-1, C-2 and C-3, respectively. The highest total epigallocatechin (1150 µg/g) and epicatechin (1510 µg/g) values were detected in the cookie of C-4. As indicated above, the sample of C-4 had the highest total scavenging activities against both radicals. It was very likely that matcha tea was the main flavonoid source in the sample of C-4. Komes et al. reported that matcha tea was rich in epigallocatechin and epicatechin [34]. Lastly, the highest total rutin (306 µg/g) and resveratrol (8.35 µg/g) contents were found in the cookie of C-5. The highest total flavonoids and stilbene contents in free and bound phenolic compounds were observed in the cookie of C-4; on the other hand, the control cookie had the lowest values.

Table 6 shows the variable composition of 15 phenolic acids investigated in this study. When compared to control sample, the C-1 cookie had the highest total chlorogenic acid (14.3 µg/g), neochlorogenic acid (183 µg/g) and caffeic acid (6.73 µg/g), sample C-2, gallic acid (78.7 µg/g), *p*-hydroxybenzoic acid (9.02 µg/g) and *o*-coumaric acid (5.48 µg/g) contents and sample C-5, the highest total ferulic acid (113 µg/g). C-4 was the richest one with the highest total of vanillic (609 µg/g), sinapic (97.8 µg/g), syringic (39.6 µg/g), ellagic (30.0 µg/g), protocatechuic (20.4 µg/g), and protocatechuic ethyl acids (28.1 µg/g). Koláčková et al. showed that sinapic acid was one of the most important phenolic acids in matcha tea, but its content in cookies was five times higher than that of their study [29]. Their research also estimated that chlorogenic acid had the most abundant phenolic acid in matcha, but in sample C-4 it was presented only in small amounts; however, neochlorogenic acid content in these cookies was approximately 22× higher than that of the content of their research. It can also be indicated that neochlorogenic acid was present in all cookies in significant amounts (55.2 to 183 μg/g), as well as gallic acid (17.3 to 78.7 μg/g) and vanillic acid (1.43 to 609 μg/g). These phenolic acids were the most abundant in all cookies.

Free flavonoids and stilbenes form 71 to 89%, and free phenolic acids 61 to 94% of the total phenolic compounds determined in all cookies. The ratio of free to bound phenolic compounds can be affected by the baking process, where high temperatures can break up the bonds connected to other cell components and result in an increase in free phenolic compounds [35]. In each cookie sample, the first five most abundant compounds in total were the same as first five compounds in free forms. In addition, most of the phenolic compounds were presented in free form; only in a few cases did they predominate in bound form, e.g., kaempferol (56–100%, except for sample C-4, Table 5). Even when the free form predominated, values of epigallocatechin and epicatechin in bound phenolic fraction of sample C-4 were still significant (246 and 420 μg/g, respectively, Table 5), as well as vanillic acid in the cookies of C-4 and C-2 (231 and 137 μg/g, respectively, Table 6). Selected chromatograms are displayed in Figures S1–S4.

The correlations between radical scavenging activities and some phenolic compound contents are presented in Table 7. The correlations exhibited that total vanillic, protocatechuic, syringic, sinapic and ellagic acid contents showed strong positive correlations (>0.91) with the DPPH scavenging activity even if their amount in the cookies was not very high. In case of total protocatechuic, vanillic, syringic and sinapic acid contents, they displayed strong positive correlations (>0.91) with the ABTS scavenging activity in this study.

## 4. Conclusions

Ingredients such as uncommon cereal flours, edible flowers, dried fruits, matcha or nuts are preferred to be incorporated into cookies in order to increase the nutritional value and popularity of the product. As previously reported in the literature (these ingredients not analysed individually in this study), these newly ingredients include valuable bioactive substances based on polyphenols and other antioxidant-rich compounds. The enrichment of cookies led to an increase in the fibre, ash and total phenolic compound contents as well as antioxidant activity properties. The main result of enrichment was the decrease in starch content and the reduction in the digestibility of the cookies. The protein contents of some samples also decreased, and it could be improved to replace with protein isolates in the future. Matcha tea seems to be the most promising ingredient to enrich cookies in terms of phenolics. Concerning individual phenolic compounds, they were mainly presented in the free form in all cookies. Although their profiles slightly differed for every cookie sample, neochlorogenic, gallic and vanillic acids were the most abundant phenolics in all cookies. The cookie recipe including kamut, jasmine, dried mango and matcha tea (the C-4 cookie) was extremely rich in flavonoids, specifically epicatechin and epigallocatechin. It can be recommended to the bakery industry as an unconventional cookie due to its health benefits to consumers.

## 5. Patents

Utility model No. 33 013, 2019. Sumczynski, D.; Šťastná, K.; Burešová, I.; Koláčková, T. The mixture for the production of cookies and durably pastries with addition of nutraceutical raw materials. Industrial Property Office of the Czech Republic, Prague, Czech Republic.

## Figures and Tables

**Table 1:** Recipe of the cookies supplemented with uncommon cereal flours, edible flower and dried fruit flours.

Ingredients (%, *w*/*w*)	Control	C-1	C-2	C-3	C-4	C-5
Wheat flour	100	18.0	43.0	59.3	43.0	40.8
Substitute flour	-	82.0 Whole Spelt	57.0 Oat flakes	40.7 Barley flakes	57.0 Kamut	59.2 Red wheat
Edible flowers	-	0.8 Lavender	3.6 Rose	1.2 Hop flower	4.1 Jasmine	3.4 Elderflower
Flavouring ingredients	-	44.5 Dried apricot	3.3 Raspberry ^1^	16.5 Dried malt extract	43.0 Dried mango	2.8 Dried lemon peel
			5.5 Strawberry ^1^	14.3 Dried ginger	5.8 Matcha tea	22.4 Dried apple
			18.2 Cashew			
Butter	61.0	62.2	69.5	61.8	69.8	78.2
Powdered sugar	32.6	33.4	37.3	16.5	37.2	41.9
Egg yolk	17.5	17.8	20.2	17.7	19.9	22.1
Vanillin sugar	6.5	6.7	7.5	6.6	7.4	8.4
Salt	0.1	0.1	0.1	0.1	0.1	0.1

^1^ Freeze-dried (lyophilised).

**Table 2:** Chemical compositions and in vitro digestibility values of the cookies.

Nutritional Properties	Control	C-1	C-2	C-3	C-4	C-5
DM (%)	98.0 ± 0.1 ^a^	94.7 ± 0.2 ^b^	96.8 ± 0.1 ^c^	96.2 ± 0.1 ^d^	95.6 ± 0.2 ^e^	96.1 ± 0.1 ^c^
CP (%)	10.1 ± 0.3 ^a^	11.0 ± 0.3 ^b^	10.2 ± 0.3 ^a^	8.4 ± 0.5 ^c^	8.6 ± 0.4 ^c^	9.4 ± 0.3 ^d^
CFat (%)	28.3 ± 0.2 ^a^	25.0 ± 0.2 ^b^	30.8 ± 1.3 ^c^	27.2 ± 0.6 ^d^	22.6 ± 1.7 ^e^	28.0 ± 0.1 ^f^
Ash (%)	0.56 ± 0.03 ^a^	1.44 ± 0.04 ^b^	1.12 ± 0.03 ^c^	0.80 ± 0.05 ^d^	0.97 ± 0.01 ^e^	1.06 ± 0.01 ^f^
TCH (%)	63.2 ± 0.2 ^a^	68.1 ± 0.2 ^b^	61.1 ± 0.5 ^c^	67.6 ± 0.4 ^d^	72.5 ± 0.7 ^e^	65.6 ± 0.1 ^f^
Starch (%)	43.6 ± 1.5 ^a^	34.2 ± 1.4 ^b^	29.3 ± 1.5 ^c^	41.6 ± 1.5 ^d^	34.4 ± 0.4 ^b^	34.6 ± 0.7 ^b^
CF (%)	1.17 ± 0.13 ^a^	1.95 ± 0.05 ^b^	1.92 ± 0.12 ^b^	1.51 ± 0.15 ^c^	2.00 ± 0.04 ^d^	1.62 ± 0.12 ^e^
NDF (%)	1.88 ± 0.16 ^a^	4.53 ± 0.26 ^b^	4.76 ± 0.19 ^c^	2.64 ± 0.19 ^d^	4.99 ± 0.16 ^e^	4.70 ± 0.01 ^c^
OMD (%)	99.2 ± 0.1 ^a^	95.3 ± 0.3 ^b,d^	95.4 ± 0.5 ^c,b^	97.2 ± 0.1 ^d^	96.0 ± 0.4 ^e^	96.4 ± 0.5 ^e^
DMD (%)	98.3 ± 0.3 ^a^	94.1 ± 0.3 ^b,d^	94.4 ± 0.5 ^c,b^	96.2 ± 0.1 ^d^	94.9 ± 0.4 ^e^	95.3 ± 0.5 ^e^
Energy (kJ/100 g)	2245 ± 15 ^a^	2148 ± 14 ^b^	2282 ± 20 ^c^	2210 ± 34 ^d^	2110 ± 29 ^e^	2221 ± 11 ^d^

Results are presented as means ± SD (*n* = 5) on dry weight basis. Values followed by the different letters in the same line are significantly different (*p* < 0.05). DM—dry matter; CP—crude protein; CFat—crude fat; TCH—total carbohydrates; CF—crude fibre; NDF—neutral-detergent fibre; OMD—organic matter digestibility; DMD—dry matter digestibility.

**Table 3:** Free and bound phenolic compound (PC) contents and antioxidant scavenging activities of each fraction of the cookies.

		Control	C-1	C-2	C-3	C-4	C-5
Free PC	mg GAE/g	0.47 ± 0.02 ^a^ 46%	1.22 ± 0.03 ^b^ 70%	1.68 ± 0.09 ^c^ 52%	1.28 ± 0.11 ^b^ 68%	3.24 ± 0.13 ^d^ 68%	1.61 ± 0.05 ^c^ 72%
Bound PC		0.54 ± 0.10 ^a^ 54%	0.52 ± 0.01 ^a^ 30%	1.56 ± 0.06 ^b^ 48%	0.61 ± 0.01 ^c^ 32%	1.52 ± 0.08 ^b^ 32%	0.62 ± 0.01 ^c^ 28%
Total PC		1.01 ± 0.08 ^a^	1.74 ± 0.01 ^b^	3.24 ± 0.04 ^c^	1.90 ± 0.06 ^d^	4.76 ± 0.16 ^e^	2.22 ± 0.03 ^f^
DPPH: Free PC	mg TE/g	0.19 ± 0.01 ^a^ 40%	0.72 ± 0.03 ^b^ 53%	2.33 ± 0.13 ^c^ 68%	0.86 ± 0.07 ^d^ 57%	4.79 ± 0.15 ^e^ 84%	1.24 ± 0.04 ^f^ 59%
DPPH: Bound PC		0.28 ± 0.03 ^a^ 60%	0.64 ± 0.04 ^b^ 47%	1.12 ± 0.09 ^c^ 32%	0.64 ± 0.04 ^b^ 43%	0.88 ± 0.07 ^d^ 16%	0.86 ± 0.04 ^d^ 41%
DPPH: Total PC		0.47 ± 0.02 ^a^	1.36 ± 0.03 ^b^	3.45 ± 0.11 ^c^	1.50 ± 0.06 ^d^	5.67 ± 0.08 ^e^	2.10 ± 0.04 ^f^
ABTS: Free PC	mg TE/g	0.51 ± 0.01 ^a^ 41%	1.53 ± 0.08 ^b^ 49%	4.30 ± 0.14 ^c^ 54%	1.97 ± 0.14 ^d^ 56%	10.6 ± 1.0 ^e^ 78%	2.42 ± 0.13 ^f^ 67%
ABTS: Bound PC		0.74 ± 0.08 ^a^ 59%	1.58 ± 0.20 ^b^ 51%	3.74 ± 0.09 ^c^ 46%	1.58 ± 0.10 ^b^ 44%	3.03 ± 0.18 ^d^ 22%	1.03 ± 0.06 ^e^ 33%
ABTS: Total PC		1.25 ± 0.05 ^a^	3.11 ± 0.09 ^b^	8.04 ± 0.11 ^c^	3.55 ± 0.12 ^d^	13.6 ± 1.2 ^e^	3.45 ± 0.10 ^d^
PCL-ACW	mg AAE/g	0.04 ± 0.01 ^a^	0.29 ± 0.02 ^b^	0.34 ± 0.01 ^c^	0.10 ± 0.04 ^d^	0.63 ± 0.02 ^e^	0.81 ± 0.04 ^f^
PCL-ACL	mg TE/g	0.06 ± 0.01 ^a^	0.54 ± 0.05 ^b^	1.39 ± 0.01 ^c^	0.44 ± 0.03 ^d^	3.19 ± 0.22 ^e^	1.45 ± 0.01 ^f^

Results are presented on dry weight basis as mean ± SD (*n* = 5). Values followed by the different letters in the same line are significantly different (*p* < 0.05). The percentage contributions of free and bound fractions to the total phenolic contents or to the total antioxidant activities are presented. PCL-ACW: antioxidant activity of water-soluble compounds measured using PCL method; PCL-ACL: antioxidant activity of lipid soluble compounds measured using PCL method.

**Table 4:** Correlation coefficients between scavenging activities and free, bound and total phenolic compound (TPC) contents.

	*r* ^1^	
DPPH	Free PC	0.9672
	Bound PC	0.7464
	Total PC	0.9993
ABTS	Free PC	0.9683
	Bound PC	0.9446
	Total PC	0.9899

^1^ Pearson’s correlation coefficient; *p* < 0.05.

**Table 5:** Flavonoid and stilbene contents of free and bound phenolic compounds extracted from the cookies.

μg/g		Control	C-1	C-2	C-3	C-4	C-5
Epigallocatechin	Free	ND	0.50 ± 0.04 ^a^	56.2 ± 0.6 ^b^	42.7 ± 2.8 ^c^	901 ± 11 ^d^	73.4 ± 1.2 ^e^
	Bound	ND	ND	8.54 ± 0.5 ^a^	ND	246 ± 4 ^b^	12.1 ± 0.3 ^c^
	Total FS	ND	0.50 ± 0.04 ^a^	64.7 ± 0.6 ^b^	42.7 ± 2.8 ^c^	1150 ± 8 ^d^	85.6 ± 0.8 ^e^
Catechin	Free	1.35 ± 0.04 ^a^	43.2 ± 2.8 ^b^	23.7 ± 0.7 ^c^	2.96 ± 0.14 ^d^	5.67 ± 0.21 ^e^	4.14 ± 0.21 ^f^
	Bound	1.57 ± 0.05 ^a^	3.51 ± 0.32 ^b^	1.29 ± 0.14 ^c^	0.81 ± 0.04 ^d^	1.59 ± 0.14 ^a^	4.43 ± 0.26 ^e^
	Total FS	2.92 ± 0.05 ^a^	46.7 ± 1.56 ^b^	25.0 ± 0.4 ^c^	3.77 ± 0.09 ^d^	7.16 ± 0.18 ^e^	8.57 ± 0.24 ^f^
Epicatechin	Free	4.90 ± 0.12 ^a^	4.17 ± 0.07 ^b^	5.79 ± 0.01 ^c^	1.91 ± 0.11 ^d^	1090 ± 29 ^e^	22.1 ± 0.1 ^f^
	Bound	0.97 ± 0.04 ^a^	0.62 ± 0.04 ^b^	11.5 ± 0.3 ^c^	ND	420 ± 4 ^d^	19.3 ± 0.1 ^e^
	Total FS	5.87 ± 0.08 ^a^	4.79 ± 0.06 ^b^	17.3 ± 0.14 ^c^	1.91 ± 0.11 ^d^	1510 ± 17 ^e^	41.4 ± 0.1 ^f^
Rutin	Free	0.09 ± 0.01 ^a^	1.27 ± 0.10 ^b^	153 ± 1 ^c^	0.59 ± 0.01 ^d^	4.79 ± 0.24 ^e^	266 ± 1 ^f^
	Bound	ND	ND	4.94 ± 0.9 ^a^	ND	0.24 ± 0.02 ^b^	40.4 ± 0.06 ^c^
	Total FS	0.09 ± 0.01 ^a^	1.27 ± 0.10 ^b^	158 ± 1 ^c^	0.59 ± 0.01 ^d^	5.03 ± 0.13 ^e^	306 ± 1 ^f^
Quercetin	Free	ND	ND	ND	ND	ND	ND
	Bound	ND	1.46 ± 0.05 ^a^	ND	3.78 ± 0.01 ^b^	ND	ND
	Total FS	ND	1.46 ± 0.05 ^a^	ND	3.78 ± 0.01 ^b^	ND	ND
Kaempferol	Free	ND	0.32 ± 0.02 ^a^	3.49 ± 0.20 ^b^	ND	0.52 ± 0.04 ^c^	ND
	Bound	ND	5.72 ± 0.05 ^a^	4.35 ± 0.23 ^b^	3.01 ± 0.04 ^c^	ND	0.43 ± 0.02 ^d^
	Total FS	ND	6.04 ± 0.03 ^a^	7.84 ± 0.41 ^b^	3.01 ± 0.04 ^c^	0.52 ± 0.04 ^d^	0.43 ± 0.02 ^e^
Resveratrol	Free	ND	1.41 ± 0.01 ^a^	2.63 ± 0.05 ^b^	1.98 ± 0.03 ^c^	1.64 ± 0.05 ^d^	8.35 ± 0.04 ^e^
	Bound	ND	ND	0.40 ± 0.01 ^a^	1.06 ± 0.01 ^b^	2.45 ± 0.15 ^c^	ND
	Total FS	ND	1.41 ± 0.01 ^a^	3.03 ± 0.03 ^b^	3.03 ± 0.02 ^b^	4.09 ± 0.10 ^c^	8.35 ± 0.04 ^d^
Free FS		6.34 ± 0.17 ^a^	50.9 ± 3.1 ^b^	245 ± 3 ^c^	50.1 ± 3.1 ^b^	2010 ± 42 ^d^	374 ± 3 ^e^
Bound FS		2.54 ± 0.15 ^a^	11.3 ± 0.5 ^b^	31.0 ± 3.1 ^c^	8.66 ± 0.10 ^d^	670 ± 8 ^e^	76.7 ± 1.6 ^f^
Total FS		8.88 ± 0.32 ^a^	62.2 ± 3.6 ^b^	276 ± 6 ^c^	58.8 ± 3.2 ^b^	2680 ± 50 ^d^	451 ± 5 ^e^

Results are presented as means ± SD (*n* = 5) on dry weight basis. Values followed by the different letters in the same line are significantly different (*p* < 0.05). ND = not detected. Free FS: flavonoids and stilbenes in free phenolic fraction; bound FS: flavonoids and stilbenes in bound phenolic fraction; total FS: total flavonoids and stilbenes.

**Table 6:** Phenolic acid contents of free and bound phenolic compounds extracted from the cookies.

μg/g		Control	C-1	C-2	C-3	C-4	C-5
Chlorogenic acid	Free	ND	8.94 ± 0.17 ^a^	4.49 ± 0.06 ^b^	1.29 ± 0.05 ^c^	5.88 ± 0.14 ^d^	2.93 ± 0.12 ^e^
	Bound	ND	5.31 ± 0.59 ^a^	1.82 ± 0.18 ^b^	1.70 ± 0.22 ^b^	ND	0.69 ± 0.06 ^c^
	Total PC	ND	14.3 ± 0.6 ^a^	6.31 ± 0.22 ^b^	2.99 ± 0.13 ^c^	5.88 ± 0.14 ^d^	3.62 ± 0.09 ^e^
Gallic acid	Free	17.3 ± 0.3 ^a^	26.2 ± 0.6 ^b^	73.0 ± 0.1 ^c^	76.9 ± 0.9 ^d^	36.6 ± 1.4 ^e^	26.4 ± 0.4 ^b^
	Bound	0.04 ± 0.01 ^a^	0.79 ± 0.02 ^b^	5.74 ± 0.01 ^c^	0.76 ± 0.01 ^b^	6.46 ± 0.02 ^d^	0.84 ± 0.06 ^b^
	Total PC	17.3 ± 0.2 ^a^	27.0 ± 0.3 ^b^	78.7 ± 0.1 ^c^	77.7 ± 0.5 ^d^	43.1 ± 0.7 ^e^	27.2 ± 0.2 ^b^
Protocatechuic acid	Free	2.04 ± 0.05 ^a^	7.40 ± 0.16 ^b^	15.9 ± 0.9 ^c^	6.98 ± 0.31 ^b^	17.7 ± 0.6 ^d^	6.42 ± 0.19 ^e^
	Bound	ND	2.90 ± 0.16 ^a^	1.97 ± 0.04 ^b^	2.56 ± 0.24 ^a^	2.71 ± 0.03 ^a^	1.77 ± 0.01 ^c^
	Total PC	2.04 ± 0.05 ^a^	10.3 ± 0.16 ^b^	17.9 ± 0.9 ^c^	9.54 ± 0.55 ^b^	20.4 ± 0.6 ^d^	8.19 ± 0.1 ^e^
Neochlorogenic acid	Free	48.2 ± 0.2 ^a^	165 ± 1 ^b^	104 ± 1 ^c^	76.1 ± 1.2 ^d^	106 ± 3 ^c^	83.8 ± 0.2 ^e^
	Bound	7.00 ± 0.15 ^a^	17.9 ± 0.4 ^b,e^	19.1 ± 1.4 ^b^	12.9 ± 0.01 ^c^	23.9 ± 0.1 ^d^	16.6 ± 1.2 ^e^
	Total PC	55.2 ± 0.2 ^a^	183 ± 1 ^b^	123 ± 1 ^c^	89.0 ± 0.6 ^d^	130 ± 2 ^e^	100 ± 1 ^f^
*p*-hydroxybenzoic acid	Free	0.65 ± 0.07 ^a^	0.82 ± 0.01 ^b^	8.17 ± 0.21 ^c^	1.06 ± 0.03 ^d^	2.29 ± 0.12 ^e^	1.16 ± 0.03 ^f^
	Bound	ND	0.51 ± 0.01 ^a^	0.85 ± 0.05 ^b^	0.14 ± 0.01 ^c^	4.43 ± 0.01 ^d^	ND
	Total PC	0.65 ± 0.07 ^a^	1.33 ± 0.01 ^b^	9.02 ± 0.13 ^c^	1.20 ± 0.02 ^d^	6.72 ± 0.07 ^e^	1.16 ± 0.03 ^d^
Vanillic acid	Free	26.2 ± 0.3 ^a^	17.8 ± 0.2 ^b^	35.5 ± 0.5 ^c^	1.11 ± 0.06 ^d^	378 ± 9 ^e^	97.4 ± 1.7 ^f^
	Bound	ND	1.11 ± 0.02 ^a^	137 ± 0.01 ^b^	0.32 ± 0.03 ^c^	231 ± 1 ^d^	1.72 ± 0.04 ^e^
	Total PC	26.2 ± 0.3 ^a^	18.9 ± 0.1 ^b^	173 ± 0.3 ^c^	1.43 ± 0.05 ^d^	609 ± 5 ^e^	99.1 ± 0.9 ^f^
Caffeic acid	Free	ND	6.43 ± 0.42 ^a^	0.19 ± 0.01 ^b^	0.76 ± 0.02 ^c^	0.98 ± 0.02 ^d^	0.75 ± 0.03 ^c^
	Bound	ND	0.30 ± 0.01 ^a^	ND	0.27 ± 0.01 ^b^	ND	0.06 ± 0.01 ^c^
	Total PC	ND	6.73 ± 0.21 ^a^	0.19 ± 0.01 ^b^	1.03 ± 0.01 ^c^	0.98 ± 0.02 ^d^	0.81 ± 0.02 ^e^
Syringic acid	Free	0.12 ± 0.02 ^a^	6.65 ± 0.90 ^b^	2.57 ± 0.08 ^c^	0.14 ± 0.01 ^a^	27.0 ± 0.3 ^d^	3.24 ± 0.13 ^e^
	Bound	ND	0.67 ± 0.01 ^a^	12.1 ± 0.2 ^b^	ND	12.6 ± 0.2 ^c^	0.13 ± 0.01 ^d^
	Total PC	0.12 ± 0.02 ^a^	7.32 ± 0.5 ^b^	14.7 ± 0.1 ^c^	0.14 ± 0.01 ^a^	39.6 ± 0.3 ^d^	3.37 ± 0.07 ^e^
*p*-coumaric acid	Free	0.33 ± 0.01 ^a^	3.66 ± 0.17 ^b^	0.74 ± 0.03 ^c^	0.34 ± 0.01 ^a^	ND	1.67 ± 0.07 ^d^
	Bound	ND	1.10 ± 0.01 ^a^	0.23 ± 0.01 ^b^	ND	ND	0.24 ± 0.03 ^b^
	Total PC	0.33 ± 0.01 ^a^	4.76 ± 0.09 ^b^	0.97 ± 0.02 ^c^	0.34 ± 0.01 ^a^	ND	1.91 ± 0.05 ^d^
Ferulic acid	Free	11.5 ± 0.8 ^a^	5.70 ± 0.10 ^b^	29.3 ± 0.6 ^c^	0.52 ± 0.02 ^d^	17.5 ± 0.5 ^e^	111 ± 1 ^f^
	Bound	0.99 ± 0.07 ^a^	0.07 ± 0.01 ^b^	11.4 ± 0.4 ^c^	ND	0.84 ± 0.04 ^d^	2.17 ± 0.01 ^e^
	Total PC	12.5 ± 0.9 ^a^	5.77 ± 0.06 ^b^	40.7 ± 0.5 ^c^	0.52 ± 0.02 ^d^	18.3 ± 0.3 ^e^	113 ± 1 ^f^
Sinapic acid	Free	ND	0.17 ± 0.01 ^a^	26.4 ± 1.0 ^b^	0.63 ± 0.04 ^c^	86.1 ± 1.5 ^d^	0.69 ± 0.03 ^c^
	Bound	ND	ND	7.34 ± 0.12 ^a^	0.16 ± 0.02 ^b^	11.7 ± 0.7 ^c^	0.61 ± 0.05 ^d^
	Total PC	ND	0.17 ± 0.01 ^a^	33.7 ± 0.6 ^b^	0.79 ± 0.03 ^c^	97.8 ± 1.5 ^d^	1.30 ± 0.04 ^e^
Ellagic acid	Free	ND	2.22 ± 0.14 ^a^	12.4 ± 0.1 ^b^	0.67 ± 0.04 ^c^	30.0 ± 0.1 ^d^	19.5 ± 0.2 ^e^
	Bound	ND	ND	5.43 ± 0.16 ^a^	4.53 ± 0.20 ^b^	ND	0.50 ± 0.01 ^c^
	Total PC	ND	2.22 ± 0.14 ^a^	17.8 ± 0.5 ^b^	5.20 ± 0.12 ^c^	30.0 ± 0.1 ^d^	20.0 ± 0.1 ^e^
*o*-coumaric acid	Free	ND	0.49 ± 0.01 ^a^	5.48 ± 0.15 ^b^	0.77 ± 0.02 ^c^	0.18 ± 0.01 ^d^	0.23 ± 0.01 ^e^
	Bound	ND	ND	ND	0.07 ± 0.01	ND	ND
	Total PC	ND	0.49 ± 0.01 ^a^	5.48 ± 0.15 ^b^	0.84 ± 0.01 ^c^	0.18 ± 0.01 ^d^	0.23 ± 0.01 ^e^
Protocatechuic ethyl acid	Free	0.88 ± 0.01 ^a^	2.06 ± 0.09 ^b^	0.81 ± 0.01 ^c^	0.08 ± 0.01 ^d^	22.0 ± 0.1 ^e^	1.96 ± 0.10 ^f^
	Bound	ND	0.25 ± 0.02 ^a^	0.05 ± 0.01 ^b^	ND	6.07 ± 0.04 ^c^	ND
	Total PC	0.88 ± 0.01 ^a^	2.31 ± 0.06 ^b^	0.86 ± 0.01 ^c^	0.08 ± 0.01 ^d^	28.1 ± 0.1 ^e^	1.96 ± 0.10 ^f^
Cinnamic acid	Free	0.01 ± 0.01 ^a^	0.31 ± 0.01 ^b^	0.16 ± 0.01 ^c^	0.03 ± 0.01 ^d^	0.14 ± 0.01 ^e^	1.34 ± 0.02 ^f^
	Bound	ND	ND	0.03 ± 0.01 ^a^	ND	0.04 ± 0.01 ^a^	0.04 ± 0.01 ^a^
	Total PC	0.01 ± 0.01 ^a^	0.31 ± 0.01 ^b^	0.19 ± 0.01 ^c^	0.03 ± 0.01 ^d^	0.18 ± 0.01 ^c^	1.38 ± 0.02 ^e^
Free PA		107 ± 2 ^a^	255 ± 3 ^b^	319 ± 5 ^c^	167 ± 3 ^d^	730 ± 17 ^e^	358 ± 4 ^f^
Bound PA		8.03 ± 0.23 ^a^	30.9 ± 1.6 ^b^	203 ± 3 ^c^	23.4 ± 0.7 ^d^	300 ± 7 ^e^	25.4 ± 1.5 ^d^
Total PA		115 ± 2 ^a^	286 ± 5 ^b^	522 ± 8 ^c^	190 ± 4 ^d^	1030 ± 20 ^e^	383 ± 6 ^f^

Results are presented as means ± SD (*n* = 5) on dry weight basis. Values followed by the different letters in the same line are significantly different (*p* < 0.05). ND = not detected. Free PA: phenolic acids in free phenolic fraction; bound PA: phenolic acids in bound phenolic fraction; total PA: total phenolic acids.

**Table 7:** Correlation coefficients between radical scavenging activities and some phenolic compound contents detected in the cookies.

	DPPH			ABTS		
*r* ^1^	Free	Bound	Total	Free	Bound	Total
Flavonoids and stilbene						
Epigallocatechin	0.9263	0.2856	0.8762	0.9577	0.4633	0.8903
Catechin	−0.1094	0.0648	−0.0770	−0.1414	−0.4191	−0.0681
Epicatechin	0.9056	0.2777	0.8547	0.9414	0.4572	0.8740
Rutin	−0.0090	0.2931	0.0630	−0.0769	−0.2934	−0.0665
Quercetin	-	−0.2269	−0.3491	-	−0.2142	−0.3091
Kaempferol	0.3081	0.2520	0.0132	0.2219	0.2859	0.0374
Resveratrol	−0.0642	0.2924	0.3492	−0.0990	0.5180	0.2213
Phenolic acids						
Chlorogenic acid	0.3700	0.0410	0.1428	0.3583	0.0028	0.1484
Gallic acid	0.1567	0.7496	0.3081	0.1153	0.9405	0.3302
Protocatechuic acid	0.9081	0.5518	0.9259	0.8712	0.4459	0.9279
Neochlorogenic acid	0.1859	0.8048	0.3179	0.1729	0.7328	0.3146
*p*-hydroxybenzoic acid	0.3768	0.3693	0.8015	0.2908	0.5998	0.8144
Vanillic acid	0.9067	0.6061	0.9450	0.9341	0.8429	0.9496
Caffeic acid	−0.1983	−0.2299	−0.2113	−0.1799	−0.3222	−0.1961
Syringic acid	0.8947	0.7126	0.9510	0.9256	0.9423	0.9681
*p*-coumaric acid	−0.4006	0.0230	−0.3387	−0.4099	−0.0848	−0.3659
Ferulic acid	−0.0642	0.6808	0.0831	−0.0380	0.6980	−0.0506
Sinapic acid	0.9774	0.6366	0.9538	0.9863	0.8516	0.9756
Ellagic acid	0.8763	0.4701	0.9166	0.8640	0.5072	0.8560
*o*-coumaric acid	0.1617	−0.1647	0.2447	0.0729	−0.1549	0.2485
Protocatechuic ethyl acid	0.8987	0.2496	0.8464	0.9341	0.4534	0.8639
Cinnamic acid	−0.0955	0.7423	−0.0132	−0.1204	0.4307	−0.1491

r ^1^ Pearson’s correlation coefficient; *p* < 0.05.

## Data Availability

Data are contained within the article.

## Supplementary Materials

The following are available online at https://www.mdpi.com/article/10.3390/foods10112531/s1, Table S1: the correlation coefficients of chemical properties with in vitro digestibility values, Figure S1: HPLC chromatogram for analysis of free phenolics in control sample; detected at the wavelength of 275 nm, Figure S2: HPLC chromatogram for analysis of bound phenolics in control sample; detected at the wavelength of 275 nm, Figure S3: HPLC chromatogram for analysis of free phenolics in kamut sample; detected at the wavelength of 275 nm, Figure S4: HPLC chromatogram for analysis of bound phenolics in kamut sample; detected at the wavelength of 275 nm.
