# Supplementary material for: Nutritional Composition, In Vitro Antioxidant Activity and Phenolic Profile of Shortcrust Cookies Supplemented by Edible Flowers
Source: Foods. 2021 Oct 21;10(11):2531. doi: 10.3390/foods10112531 (PMC8620082; doi:10.3390/foods10112531)
Supplement: Supplementary file 1 [file foods-10-02531-s001.zip › foods-1431322-supplementary.pdf]

Supplementary material

Nutritional Composition, In Vitro Antioxidant Activity and Phenolic Profile of Shortcrust Cookies Supplemented by Edible Flowers

Kristýna Šťastná [1], Daniela Sumczynski [1,*], Erkan Yalcin [2]

[1] Faculty of Technology, Department of Food Analysis and Chemistry, Tomas Bata University in Zlín, Náměstí T.G. Masaryka 5555, 760 01 Zlín, Czech Republic

[2] Faculty of Engineering, Gölköy Campus, Bolu Abant Ízzet Baysal University, Bolu 14030, Turkey

* Correspondence: sumczynski@utb.cz

Table S1. The correlation coefficients of chemical properties with in vitro digestibility values.

r[*]	CP	CFat	Ash	TCH	Starch	CF	NDF
DMD	0.1849	0.1967	0.9042	0,2534	0.8978	0.9530	0.9132
OMD	0.1924	0.2232	0.9189	0,2780	0.8795	0.9462	0.9040

[*]Pearson´s correlation coefficient.

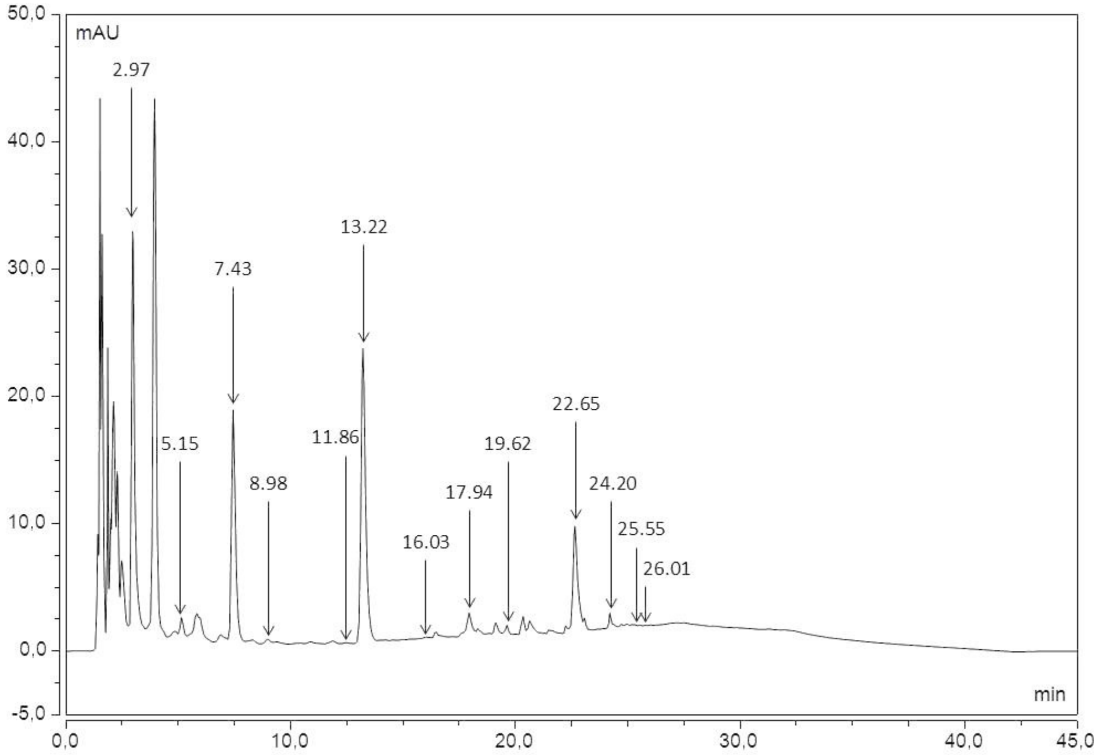

Figure S1. HPLC chromatogram for analysis of free phenolics in control sample; detected at the wavelength of 275 nm.

2.97 – gallic acid; 5.15 – protocatechuic acid; 7.43 – neochlorogenic acid; 8.98 – 4-hydroxybenzoic acid; 11.86 – catechin; 13.22 – vanillic acid; 16.03 – syringic acid; 17.94 – epicatechin; 19.62 – *trans-p*-coumaric acid; 22.65 – ferulic acid; 24.20 – protocatechuic acid ethylester; 25.55 – rutin; 26.01 – *trans*-cinnamic acid.

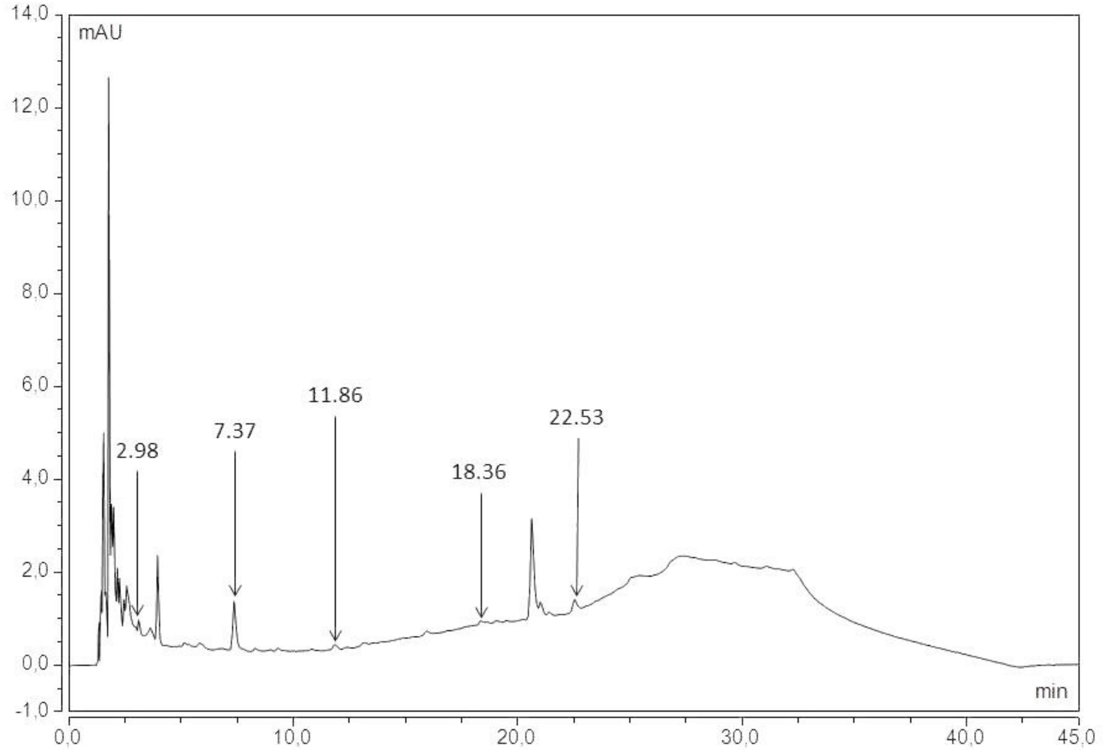

Figure S2. HPLC chromatogram for analysis of bound phenolics in control sample; detected at the wavelength of 275 nm.

2.98 – gallic acid; 7.37 – neochlorogenic acid; 11.86 – catechin; 18.36 – epicatechin; 22.53 – ferulic acid.

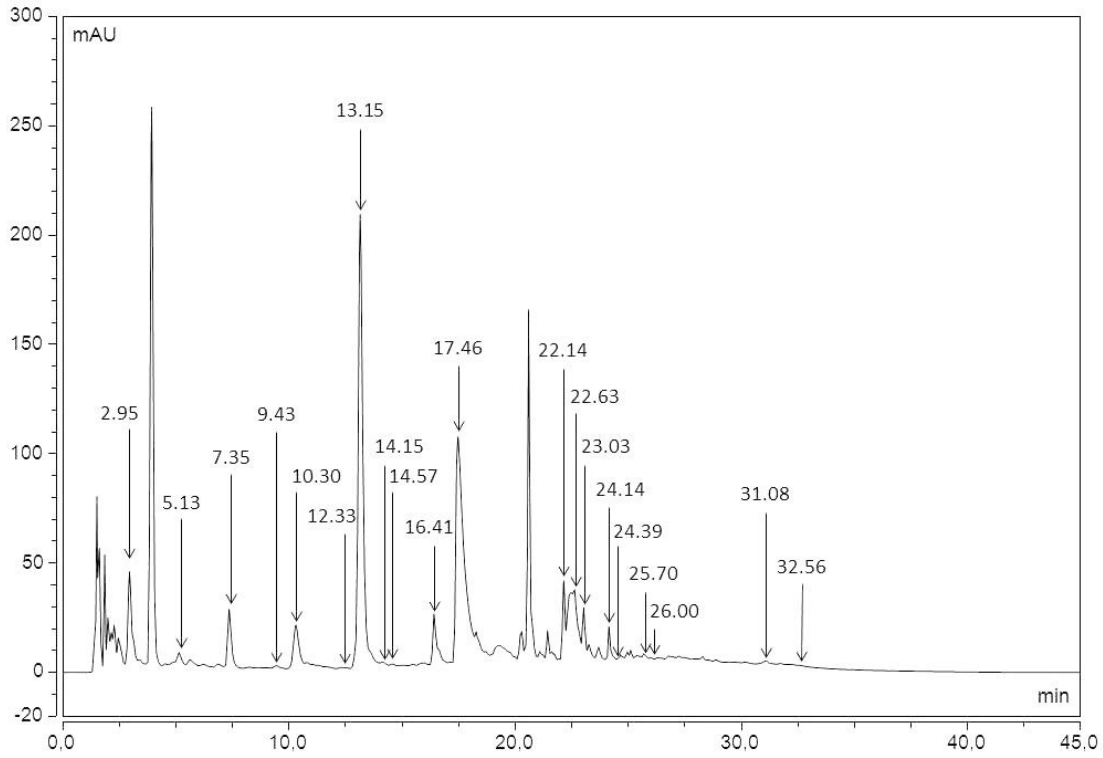

Figure S3. HPLC chromatogram for analysis of free phenolics in kamut sample; detected at the wavelength of 275 nm.

2.95 – gallic acid; 5.13 – protocatechuic acid; 7.35 – neochlorogenic acid; 9.43 – 4-hydroxybenzoic acid; 10.30 – epigallocatechin; 12.33 – catechin; 13.15 – vanillic acid; 14.15 – chlorogenic acid; 14.57 – caffeic acid; 16.41 – syringic acid; 17.46 – epicatechin; 22.14 – sinapic acid; 22.63 – ferulic acid; 23.03 – ellagic acid; 24.14 – protocatechuic acid ethylester; 24.39 – *trans*-2-hydroxycinnamic acid; 25.70 – rutin; 26.00 – *trans*-cinnamic acid; 31.08 – resveratrol; 32.56 – kaempferol.

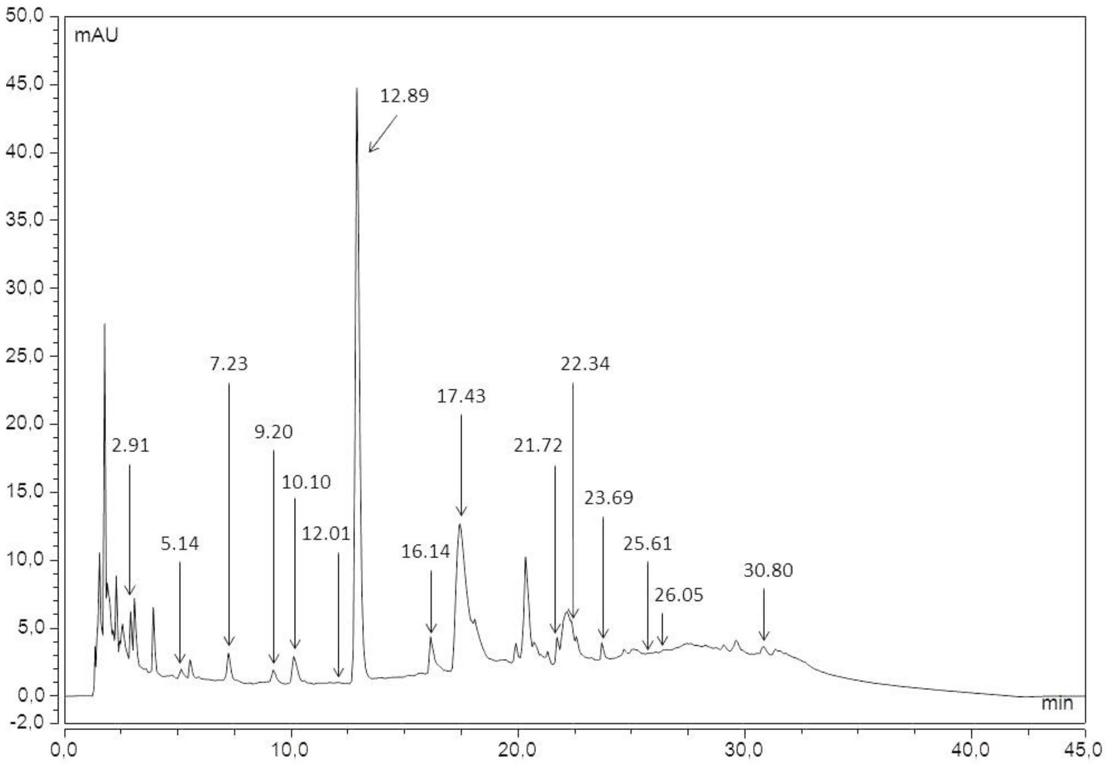

Figure S4. HPLC chromatogram for analysis of bound phenolics in kamut sample; detected at the wavelength of 275 nm.

2.91 – gallic acid; 5.14 – protocatechuic acid; 7.23 – neochlorogenic acid; 9.20 – 4-hydroxybenzoic acid; 10.10 – epigallocatechin; 12.01 – catechin; 12.89 – vanillic acid; 16.14 – syringic acid; 17.43 – epicatechin; 21.72 – sinapic acid; 22.34 – ferulic acid; 23.69 – protocatechuic acid ethylester; 25.61 – rutin; 26.05 – *trans*-cinnamic acid; 30.80 – resveratrol.
